# Rethinking Dementia Awareness Training: How Elapsed Time Shapes Knowledge Retention among Businesses

**DOI:** 10.1093/geroni/igaf122.2340

**Published:** 2025-12-31

**Authors:** Elena Ionescu, Monit Cheung

**Affiliations:** California State University, Long Beach, Houston, Texas, United States; University of Houston, Houston, Texas, United States

## Abstract

Dementia-Friendly Businesses (DFBs) play a crucial role in fostering inclusive environments for individuals living with dementia. While workforce training aims to enhance dementia-friendly awareness, little research has examined how training mode or time elapsed since training may influence long-term knowledge retention and behavioral adaptation. This study assessed dementia knowledge retention among 510 employees in DFB settings, evaluating whether training mode or time elapsed had a greater impact on knowledge retention for longer-term dementia-friendly awareness. Guided by the Theory of Planned Behavior (TBP) and Dementia-Centered Person Care (DCPC), findings show that the training mode does not significantly vary workforce dementia awareness. A two-way ANOVA examined the effects of training mode and time elapsed on dementia awareness scores. The training mode was insignificant (F(2, 501) = .709, p = .492), suggesting that in-person, hybrid, and online training were equally effective. However, time elapsed significantly impacted awareness (F(2, 501) = 17.547, p < .001), with employees trained over a year ago exhibiting higher scores (M = 132.93, SE = 1.43) than those trained 6–12 months ago (M = 128.46, SE = 1.27, p < .001) or < 6 months ago (M = 119.89, SE = 1.68, p < .001). Post-hoc analyses confirmed that awareness increases over time, regardless of training mode. Findings reinforce the importance of sustained engagement and exposure in strengthening dementia awareness, aligning with the TBP’s emphasis on sustained normative influences and DCPC’s focus on continued, person-centered learning. Future research should explore optimal reinforcement strategies to sustain dementia awareness in business settings.

